# Trends in Mental Health Diagnoses Among Children and Adolescents Before and During the COVID-19 Pandemic in a Large German State

**DOI:** 10.3390/children13091245

**Published:** 2026-09-14

**Authors:** Kathrin Jobski, Michael Dörks, Alexander M. Fassmer, Imke Meißner, Jona T. Stahmeyer, Sven Cornelisse, Luise Poustka, Christian J. Bachmann, Falk Hoffmann

**Affiliations:** 1 Department of Health Services Research, Division of Outpatient Care and Pharmacoepidemiology, Carl von Ossietzky Universität Oldenburg, 26129 Oldenburg, Germany; 2 Institute for Clinical Epidemiology and Biometry, Julius-Maximilians-Universität Würzburg, 97078 Würzburg, Germany; 3 Health Services Research Unit, AOK Niedersachsen, 30519 Hannover, Germany; 4 Clinic for Child and Adolescent Psychiatry, Heidelberg University Hospital, 69120 Heidelberg, Germany; 5 Department of Child and Adolescent Psychiatry, University Hospital Ulm, 89070 Ulm, Germany

**Keywords:** COVID-19 pandemic, mental health diagnoses, children and adolescents, claims data, deprivation

## Abstract

**Highlights:**

**What are the main findings?**

- During the pandemic period the largest changes in mental health, as reflected by increased healthcare utilization, were observed for female adolescents.
- In this group mental health diagnoses (especially anxiety and depressive disorders) persisted even after most pandemic restrictions had ended.

**What are the implications of the main findings?**

- These findings highlight the critical need for sustained, targeted mental health support particularly for this demographic group.
- Further research should extend their scope into young adulthood to monitor long-term trends in mental health diagnoses and healthcare utilization.

**Abstract:**

**Background/Objectives:** During the COVID-19 pandemic, school closures and reduced peer interactions impaired the quality of life and mental health of young people. The aim of this study was to examine trends in mental health diagnoses among children and adolescents during the pandemic as compared to pre-pandemic periods in a large German state. **Methods:** We used secondary data for the period 2018–2022 from youths <18 years insured with AOK Lower Saxony (about 500,000 children and adolescents per year). Data included outpatient and hospital diagnoses, and socio-demographic information (including area deprivation). For depressive disorders, anxiety and eating disorders, as well as attention-deficit/hyperactivity disorder (ADHD), we calculated yearly incidence and prevalence, stratified by sex and age. **Results:** In 2021, the incidence was highest for anxiety disorders (34.7 per 1000 persons), followed by ADHD (18.0/1000), depressive disorders (13.7/1000), and eating disorders (2.7/1000). Compared to 2019, incidences increased for all four disorders in 2021, with changes being most pronounced in females aged 15–17 years (anxiety disorders: +23%, depressive disorders: +25%, ADHD: +54%, eating disorders: +90%), while trends were less clear for prevalences. In 2022, incidences and prevalences generally decreased but did not return to pre-pandemic values in adolescent girls. No consistent gradient across levels of area deprivation was observed. **Conclusions:** During the pandemic, we observed an increase in several mental health diagnoses as reflected by increased healthcare utilization among children and adolescents, particularly in adolescent girls. In this group the prevalence of mental health diagnoses remained high also in 2022. This underscores the necessity of providing targeted mental health support and monitoring long-term trends.

## 1. Introduction

During the COVID-19 pandemic, a declining quality of life and an increase in mental health disorders were observed among children and adolescents, with school closures and decreased peer interactions acting as triggers of psychological distress [1,2,3]. Furthermore, it is well-known that lower familial (socio)economic status and/or living in deprived neighborhoods can impact the development of mental health problems in children and adolescents [4,5]. With respect to changes in mental health, several studies focused on internalizing symptoms like anxiety and depression, whereas findings on externalizing symptoms have been more heterogeneous than the results found for internalizing symptoms [1,2]. Two meta-analyses, for example, found increases in depressive or anxiety symptoms and clinically relevant depression for European children and adolescents compared to pre-pandemic baselines, respectively, with trends varying between age groups and sex [6,7]. In both studies the effects were more pronounced in countries with higher levels of governmental response policies [6,7].

In Germany, government policies during the pandemic were rather strict as compared to other European countries or the US, e.g., in 2021 as reflected by the stringency index and the school closure index [8]. So far, previous studies and analyses based on survey or claims data reported deteriorations in well-being and mental health among children and adolescents during the pandemic [9,10,11,12]. The COPSY (COVID-19 and PSYchological Health) study, a nationwide, population-based survey conducted at five different time points, found the highest proportions of anxiety and depressive symptoms in December 2020–January 2021, with relative increases equaling 100% and 50% when compared to May–June 2020, respectively [11]. Overall, females had higher risks, as did children and adolescents belonging to a risk group including low parental education or restricted living space. Claims-based analyses reported increases in newly diagnosed depression, anxiety and eating disorders, especially among adolescent girls aged 15–17 years, with the highest values in 2021 [9,10]. These incidences tended to be higher in those living in socioeconomically disadvantaged areas; however, when comparing time trends between groups of different socioeconomic status, results were inconsistent [10]. Overall, these studies were based either on a comparatively small number of children and adolescents or mainly focused on internalizing disorders.

Therefore, the aim of the present study was to examine trends in diagnosed internalizing and externalizing mental health disorders among children and adolescents during the pandemic as compared to pre-pandemic periods in a large German territorial state. As a second exploratory aim, we investigated whether these trends varied across levels of area deprivation in adolescent girls.

## 2. Materials and Methods

### 2.1. Data Source and Study Population

We used data from the AOK Lower Saxony (AOK Niedersachsen), a statutory health insurance (SHI) provider that in 2022 insured nearly 37% of the population of the German federal state of Lower Saxony [13]. Lower Saxony is a territorial state ranking second in terms of area among the 16 German federal states and fourth with respect to population (8.1 million) [14]. In Germany, approximately 74.5 million persons (i.e., nearly 90% of the population) are insured with an SHI; this comprises around 13.5 million youths younger than 20 years [13]. Generally, patients are free to choose their physicians including many specialists although general practitioners and pediatricians, respectively, are often the first point of contact for medical issues [15,16].

The study period encompassed the years 2018 to 2022. Data included demographics for all children and adolescents younger than 18 years insured for at least one day with AOK Lower Saxony in a given year (aggregated level) as well as individual-level information (demographics, insurance times and diagnoses) for those children and adolescents living in Lower Saxony with at least one of the considered mental health diagnoses during the study period.

### 2.2. Case Definition

Given that several reviews describe increases in depressive or anxiety symptoms in children and adolescents during the pandemic [1,6,7] while trends in externalizing disorders are more heterogeneous [1], we chose a broad set of conditions, as has been done by claims-based analyses from Spain and the US [17,18]. Therefore, the subsequent mental health diagnoses were included (International Classification of Diseases and Related Health Problems, 10th revision, German Modification (ICD-10-GM)): depressive disorders (F32-, F33-, F34.0, F34.1, F41.2, F43.2, F92.0), anxiety disorders (F40-, F41-, F42-, F43-, F93-), attention-deficit/hyperactivity disorder (ADHD) (F90-, F98.8) and eating disorders (F50-). The sensitive inclusion strategy for depressive disorders, encompassing all affective disorders with a depressive component, led to partly overlapping diagnoses (i.e., mixed anxiety and depressive disorder and adjustment disorders) and persons with the respective diagnoses contributing to depressive disorders and anxiety disorders. This was chosen to account for the heterogeneous manifestation and diagnosis of pandemic-related distress (e.g., mixed anxiety-depressive states) in clinical practice.

We used diagnoses from the outpatient setting (coded with the diagnostic certainty “confirmed”) and from hospitals (main discharge diagnoses, i.e., reflecting the reason for admission). Diagnoses were assessed for each calendar year. For hospitalizations, the date of admission for the respective hospital stay was used. All four disorders were considered separately.

A prevalent case was defined as having had at least one respective diagnosis in a calendar year. Thus, an individual could qualify as a prevalent case in every year of the study period. Cases were defined as incident if they had at least one respective diagnosis in a calendar year and no diagnosis in the previous year whilst having been continuously insured in that previous year. Therefore, persons could be included as an incident case more than one time during the study period when this definition was fulfilled.

### 2.3. Case Characteristics

Case characteristics included age group, sex and area deprivation. Based on developmental stages during the transition from childhood to adolescence, age was categorized into five groups, i.e., 0–5 years (infancy and early childhood), 6–8 years (early primary school age), 9–11 years (middle primary school age), 12–14 years (early adolescence) and 15–17 years (late adolescence) [19]. Age was determined based on the individual’s age in the respective calendar year.

To account for area deprivation at the district level, we included the German Index of Multiple Deprivation (GIMD, version of 2020). The GIMD is based on seven domains representing single aspects of deprivation, which are assigned different weights (income (25%), occupation (25%), education (15%), municipal/district revenue (15%), social capital (10%), environment (5%), and security (5%)) [20]. It therefore assumes that high deprivation is not only caused by socioeconomic inequalities but also by environmental factors or inadequate infrastructure [20]. Based on all German districts (n = 401), the GIMD can be categorized into quintiles from Q1 (lowest deprivation) to Q5 (highest deprivation). For this study, the respective GIMD quintiles were assigned to both the individual-level data as well as the overall population of children and adolescents insured with AOK Lower Saxony based on the individual district of their residence. The federal state of Lower Saxony comprises different types of areas (from highly peripheral to highly central) including 37 counties and eight independent cities. With respect to the assigned GIMD quintiles, some counties bordering larger cities (e.g., in the south of Hamburg or the west of Bremen) display the lowest levels of deprivation, while Lower Saxony’s (independent) cities tend to be more deprived (e.g., Q4 for the largest district of Hannover including Hannover city).

### 2.4. Statistical Analyses

First, we calculated yearly incidences (2019–2022) per 1000 persons with 95% confidence intervals (CI) for depressive disorders, anxiety disorders, ADHD and eating disorders overall and stratified by age group and sex. Incidences (i.e., newly documented diagnoses following a 12-month diagnosis-free period) were calculated by dividing the number of incident cases in a year by all persons at risk in the respective stratum (i.e., the total population minus those with at least one respective diagnosis in the previous year). As done in a previous systematic review which examined change in antidepressant utilization in children, adolescents and young adults in Europe before and during the pandemic, we defined 2019 as the most recent pre-pandemic year, 2020 as a transitional year and 2021 as the year when the full effects of the pandemic and associated restrictions were in place [21]. For our main study question we compared incidences in 2021 (pandemic) to 2019 (pre-pandemic) by calculating relative risks (RR) with 95% CI. Accordingly, we compared the year 2022, when many restrictions were lifted, to 2019 to explore whether incidences returned to pre-pandemic values.

Second, to display general trends of mental health diagnoses, we calculated prevalences (2018–2022) overall and stratified by age group and sex for all four studied disorders. Again, we compared prevalences in 2021 to 2019 and 2022 to 2019, respectively by calculating prevalence ratios (PR) with 95% CI.

Third, since previous claims-based analyses [9,10,17,18] showed that adolescent girls were most vulnerable in terms of their mental health during the pandemic, we chose this group to further examine the potential influence of area deprivation on changes between 2019 and 2021 by stratifying incidences by the GIMD quintiles in an exploratory analysis.

Last, we reran incidence and prevalence analyses using a narrow definition for depressive disorders, i.e., including only diagnoses of depressive episode (F32-) or recurrent depressive disorder (F33-) [10].

RR and PR were calculated at the aggregate level treating each annual study population as independent cross-sectional data. CIs for incidences and prevalences were computed using the Wilson score interval method, whereas CIs for RR and PR were calculated as Wald confidence intervals.

Analyses were performed using SAS, version 9.4 (SAS Institute Inc., Cary, NC, USA).

For this study, a waiver was obtained from the Medical Ethics Committee of the Carl von Ossietzky University Oldenburg (2024-125).

## 3. Results

### 3.1. Study Population

During the study period, between 475,750 (2018) and 585,520 (2022) children and adolescents were insured with AOK Lower Saxony (Supplemental Table S1). The distribution of sex and age was very similar over time with nearly half being female, slightly more than one third aged between 0 and 5 years, while the older four age groups each accounted for about 16–17%. The distribution of GIMD quintiles was nearly identical for each year with the largest proportion of children and adolescents belonging to the fourth quintile (40%), followed by the second (23%) and third (21%) quintiles, while far fewer children and adolescents were assigned to the first (9%) and fifth quintiles (7%).

### 3.2. Incidences

Across all study years, the overall incidences were highest for anxiety disorders (ranging from 30.1 to 34.7 per 1000 persons), followed by ADHD (16.1–18.0) and depressive disorders (11.9–13.7), while the incidence of eating disorders was far lower (2.2 to 2.7 per 1000 persons, Table 1). Females consistently showed higher incidences for depressive, anxiety and eating disorders than males (factors 1.1 to 1.8), whereas ADHD incidences were substantially lower among females (factor 0.5).

When comparing the overall incidences of 2021 to those of 2019, increases were observed for all examined disorders, being most pronounced for eating disorders (+21%). In females the incidences of all disorders were significantly increased (depressive disorders: +14%, anxiety disorders: +11%, ADHD: +16%, eating disorders: +28%). Among males, a significant increase was only observed for ADHD (+8%), whereas for depressive disorders a significant decrease was observed (−10%). In 2022, incidence levels did not significantly exceed those observed in 2019; instead, significant decreases were observed for depressive and anxiety disorders (overall and in males).

**Figure 1:**
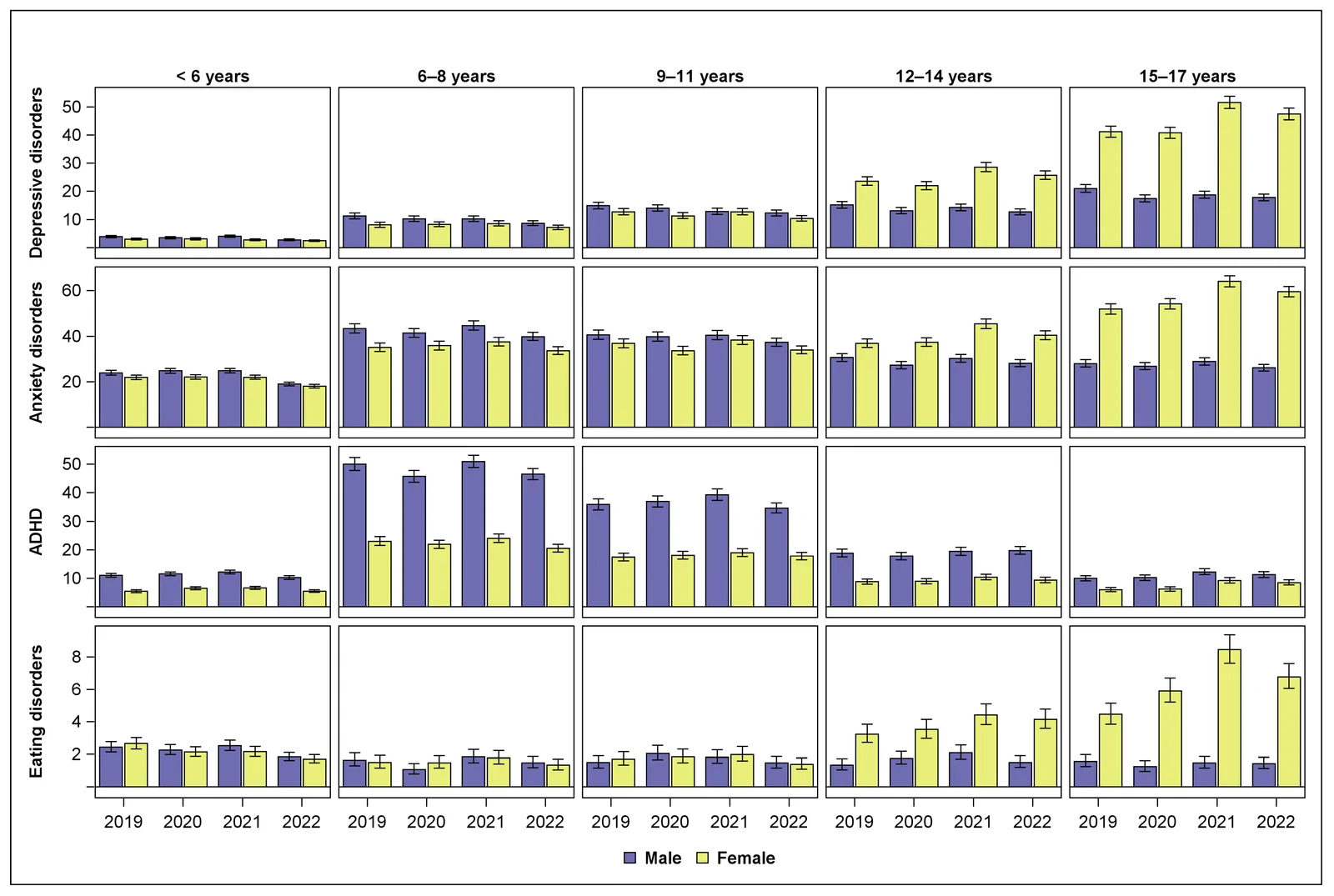
Incidence of mental health diagnoses per 1000 children and adolescents stratified by sex, age group and year. Note: The scales of the axes differ.

When considering analyses stratified by sex and age group, incidences showed far more divergent trends (Figure 1, Supplemental Table S2). In females, incidences tended to increase with age except for ADHD where the highest value was observed in those aged 6–8 years. In males, the incidence of depressive disorders was highest in those aged 15–17 years, while for anxiety disorders and ADHD the age group 6–8 years was most often affected. For eating disorders, sex differences were overall most pronounced in the age group 15–17, showing higher incidences for females for depressive disorders (factors 2 to 3), anxiety disorders (factor 2) and eating disorders (factors 3 to 6). In contrast, ADHD differences were most pronounced in the age group 6–8 with lower incidences for females (factor 0.5).

With respect to trends, for depressive disorders, incidences increased significantly in females aged 12–14 (+21%) and 15–17 years (+25%) whereas in males and younger females, decreases were found. Applying the narrow definition for depressive disorders yielded larger increases for the younger age group, i.e., +46% in females aged 12–14 years vs. +30% in those aged 15–17 years (Supplemental Table S3).

For anxiety disorders females aged 12–14 and 15–17 were the only groups with significant increases (+23% each, Figure 1, Supplemental Table S2). For ADHD, most age groups in females—except those aged 6–8 and 9–11—showed significant increases with the highest increase in those aged 15–17 (+54%). In males, significant increases were observed for those aged 0–5, 9–11 (+10% each) and 15–17 (+23%). For eating disorders, significant increases were found for females aged 12–14 (+37%) and 15–17 (+90%) and in males aged 12–14 (+58%). When comparing 2022 to 2019 changes were not significant or incidences decreased except for females aged 15–17, where every disorder displayed a significant increase, albeit smaller than the change between 2021 and 2019.

### 3.3. Area Deprivation

**Figure 2:**
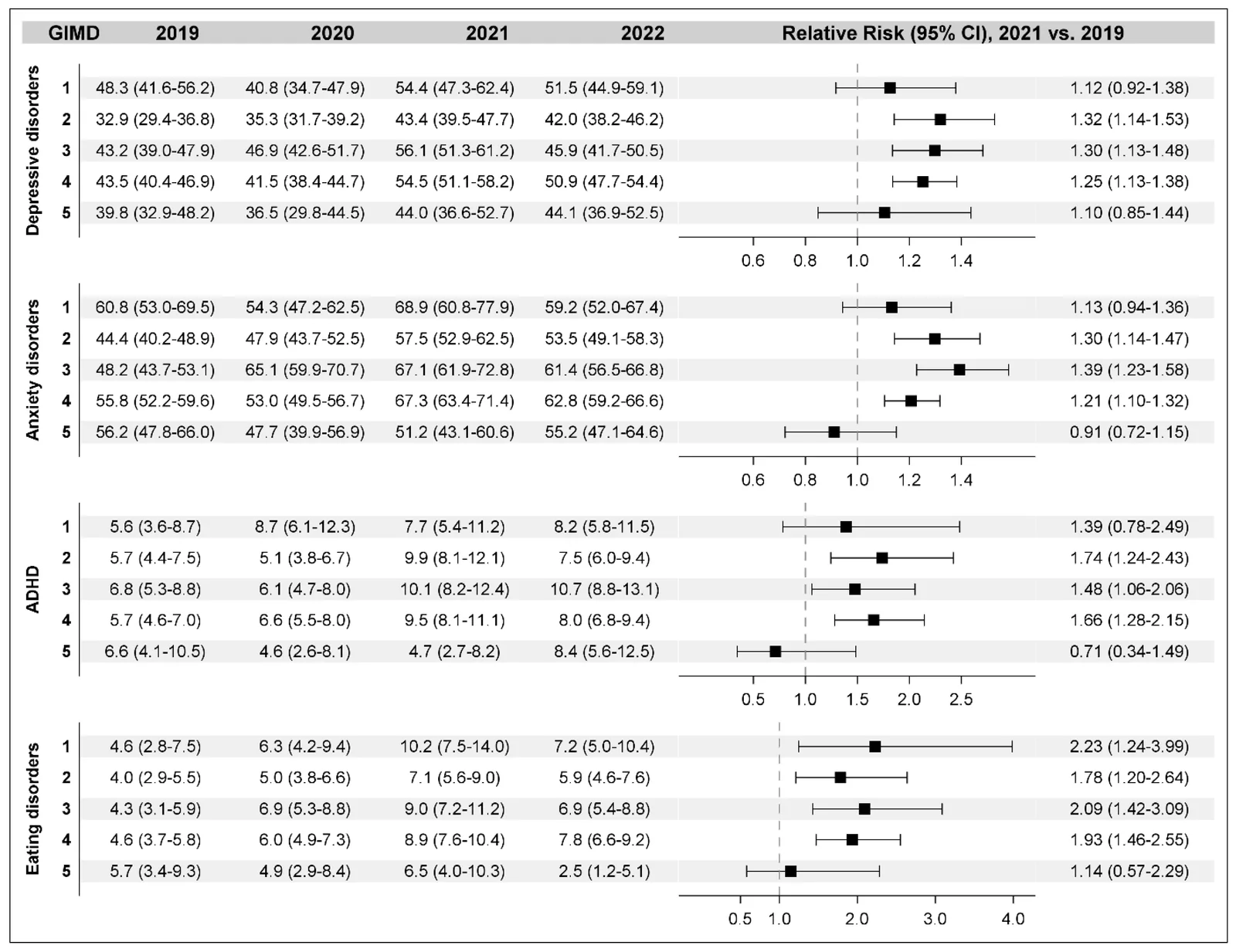
Incidence of mental health diagnoses per 1000 females aged 15–17 stratified by year and area deprivation. GIMD: German Index of Multiple Deprivation (quintiles). Note: The distribution of the GIMD quintiles in Lower Saxony is displayed in Supplemental Table S1.

The influence of area deprivation for females aged 15–17 is displayed in Figure 2. The incidence of depressive disorders in 2021 was highest in the third GIMD quintile (56.1 per 1000) and lowest in the second (43.4). Significant increases (2019–2021) were found for the GIMD quintiles 2–4 (+25 to +32%), while increases were not significant for the first (+12%) and the fifth (+10%). For anxiety disorders, incidences in 2021 were highest in the first GIMD quintile (68.9 per 1000) and lowest in the fifth (51.2) with significant increases (2019–2021) for the quintiles 2–4 (+21% to +39%), while changes were not significant for the first (+13%) and the fifth quintile (−9%). For ADHD, 2021 incidences were highest in the third GIMD quintile (10.1 per 1000) and lowest in the fifth (4.7). Significant increases were found for the quintiles 2–4 (+48% to +74%), while changes were not significant for the first (+39%) and fifth quintiles (−29%). For eating disorders, incidences in 2021 were highest in the first GIMD quintile (10.2 per 1000) and lowest in the fifth (6.5). Comparing 2021 to 2019, significant increases were found for the GIMD quintiles 1–4 with the largest increases for the first and third (+123% and +109%, respectively), while the increase was not significant for the fifth quintile (+14%).

### 3.4. Prevalences

Annual prevalences showed similar patterns to the incidences with the highest overall values for anxiety disorders (ranging from 60.3 to 66.0 per 1000 persons), followed by ADHD (50.4–56.7) and depressive disorders (24.2–26.5) with far lower values for eating disorders (3.9–4.6 per 1000 persons, Table 1). Significant increases were only observed for females and only when comparing 2021 to 2019. With respect to sex and age, prevalences for depressive, anxiety and eating disorders were highest in females aged 15–17, whereas for ADHD the highest prevalence was observed in males aged 9–11 (Supplemental Figure S1, Supplemental Table S2). For all disorders significant increases were observed in females aged 15–17 when comparing 2021 to 2019 (depressive disorders: +18%; anxiety disorders: +16%; ADHD: +22%; eating disorders: +42%). Similar patterns were found when comparing 2022 to 2019. For depressive and anxiety disorders prevalences were even higher in 2022 than in 2021.

## 4. Discussion

In our study among children and adolescents over a five-year period, the incidence of mental health diagnoses, including internalizing and externalizing disorders, increased during the pandemic, peaking in 2021. This effect could be mainly attributed to changes in females aged 15–17 years whereas males and younger females were less often affected. While newly diagnosed anxiety or depressive disorders were the overall most common diagnoses in older girls, relative changes in eating disorders were most pronounced in this group.

### 4.1. Overall Changes in Mental Health Diagnoses—Comparison to Other Data (Sources)

Although differing with respect to case definitions or age groups, very similar patterns were observed in other claims-based analyses. A study from Spain found the sharpest increases in incidences for all four disorders in 2021, being more pronounced in females, and displaying the largest increase for eating disorders when compared to 2019 [18]. In a US study, prevalences tended to be lower but revealed similar trend patterns with 13–18-year-old females experiencing an immediate increase in a pandemic period for all four disorders compared to a pre-pandemic period with eating disorders more than doubling [17]. Studies spanning larger time frames show that these increases cannot be attributed only to long-term trends [9,18]. In the case of a German analysis starting at 2014, pre-pandemic incidences showed only slight fluctuations (depressive disorders) or were on downward trends (anxiety and eating disorders) [9]. Potential reasons for the increases observed in claims-based data during the pandemic may also include structural factors such as delayed diagnoses during lockdowns followed by catch-up diagnoses or changes in diagnostic procedures as well as broader shifts such as greater mental health awareness or greater parental worry, thereby prompting more individuals to actively seek healthcare.

Survey-based studies found increases in mental health disorders in children and adolescents as well, however, displaying generally higher proportions of affected persons. The English Mental Health of Children and Young People (MHCYP) survey, e.g., observed that based on the Strengths and Difficulties Questionnaire the prevalence of a probable mental health disorder in children aged 8–16 years rose from 12.5% in 2017 to 17.7% in 2021 and to 19.0% in 2022, whereas in those aged 17–19 years, the respective proportions were 10.1% (2017), 17.4% (2021) and 25.7% (2022) [22]. Similarly, the German COPSY study based on parent- and self-reports found that during its second survey wave (December 2020/January 2021), 15.0% of children and adolescents had depressive symptoms (based on the PHQ-2) and 30.1% had anxiety symptoms (measured by SCARED), equaling increases by 50% and 100%, respectively, compared to pre-pandemic values [11]. Although proportions of affected children and adolescents decreased at the fifth survey wave (September/October 2022) to 8.6% (depressive symptoms) and 22.6% (anxiety symptoms), these values are far higher than in our study. However, it is not surprising that proportions measured by screening tools in surveys exceed those based on claims/administrative data reflecting healthcare utilization. Such differences are highly plausible and have also been observed in studies comparing both data sources [23], as claims data reflect a health system perspective that does not necessarily reflect the true disease burden. This is also in line with a recent scoping review reporting that the need for outpatient psychotherapeutic care for children and adolescents in Germany is largely unmet with the majority of treatment requests not being fulfilled and waiting times for therapy being excessively long [16].

Taking a broader perspective, the interplay between increasing demands, as reflected by the newly documented diagnoses in our study, and already strained care capacities is important to consider. During the pandemic, mental health service provision for children and adolescents changed in various directions including reduced utilization, shifts between care settings and changes in the use of psychotropic drugs [21,24,25]. These changes have also been reported for the general population, for example in a systematic review reporting substantial shifts in the utilization of mental health services, with the largest reductions in inpatient services and significant increases in telemedicine use [26].

### 4.2. Adolescent Girls—A Particularly Vulnerable Group

Across all examined disorders, adolescent females experienced the largest increases in newly documented mental health diagnoses between 2019 and 2021 with prevalence changes being less pronounced. While these trends predominantly affected those aged 15 to 17, girls aged 12 to 14 exhibited the sharpest increase regarding the narrow definition of depressive disorders which was also observed in another German claims-based analysis [10]. In comparison, younger girls and male children and adolescents showed smaller increases in diagnosed disorders. Besides potential differences in coping with the pandemic and pandemic-related restrictions, additional explanations for these findings, in more general terms, might be differences in seeking and accessing help for mental health difficulties [27].

The overall high relevance of anxiety and depressive disorders in adolescent females is well known also from pre-pandemic times as reported by parent- and self-reports in the German BELLA cohort study [23]. Further, the observed increases during the pandemic are in line with literature that identified older girls as most vulnerable especially in the context of internalizing disorders [1]. Reported reasons include an impaired social network on which girls tend to rely more than boys, increased loneliness and spending more time on social media [28,29]. Our findings are consistent with another German study which observed a sharp increase in antipsychotic drug use in 15–19-year-old females, as well as frequent diagnoses of depression and anxiety disorders/emotional disorders among this group [25].

Although eating disorders, with respect to absolute numbers, were far lower than other mental health diagnoses, corresponding to a comparatively low absolute increase of 2.3 per 1000 females, their relative increase was most pronounced. This is in line, e.g., with findings from England [22] and also with those of a meta-analysis that found that healthcare use for pediatric eating disorders increased substantially during the COVID-19 pandemic, particularly among girls and adolescents [30]. Potential explanations for these disproportionate increases are offered by a Norwegian study reporting that a rise in eating problems during the pandemic was paralleled by increases in screen time, mental distress, stress, or conflict with parents [31]. Further reasons for our findings might include increased screening or clinical recognition in adolescent females alongside pandemic-induced shifts in healthcare utilization and unequal access to specialized care.

Interestingly, females aged 15–17 also experienced the largest increase in the incidence of ADHD when comparing 2021 to 2019 and also 2022 to 2019. In contrast, for the “typical” high-risk group of males and, to a lesser extent, females in the early primary school age, no relevant increase was observed in 2021, followed by a significant decrease in 2022 as compared to 2019. However, this may reflect delayed recognition, changing diagnostic practice during the pandemic or increased clinical contact, and not necessarily a true pandemic-induced rise in the incidence. In contrast to our findings, a meta-analysis including children and adolescents between the ages of 3 and 18 years points to a global increase in ADHD symptoms but suggests that the increase in ADHD symptoms was not affected by sex or age [32].

Although in 2022 new mental health diagnoses were less common than in 2021, incidences and prevalences did not return to pre-pandemic values in females aged 15–17. In fact, for anxiety and depressive disorders, the highest disease prevalences were found in 2022, i.e., beyond the year when the full effects of the pandemic and associated restrictions were in place. As mentioned before, these numbers probably are an underestimation of the true disease burden. This underscores the necessity of providing targeted mental health support but also monitoring long-term trends.

### 4.3. Potential Influence of Area Deprivation

In our most vulnerable group of females aged 15–17, incidences and changes between 2019 and 2021 did not show a clear picture when stratified by area deprivation as measured by the GIMD at the district level. This does not seem to align with the literature, where persons with a lower socioeconomic status (SES) and/or a higher degree of deprivation overall show a higher disease burden of mental health disorders [33,34].

However, with respect to trends, studies using different deprivation measures came to inconsistent results. In the COPSY Hamburg study, e.g., comparing 2022 to 2020, children and adolescents living in more deprived areas had higher levels of depressive symptoms during the pandemic. However, the authors did not find an association between levels of neighborhood deprivation and averaged changes in mental health problems and health-related quality of life [12]. Apparently contradictory results have also been reported by an analysis using the German index of socioeconomic deprivation (GISD), a deprivation measure focusing on socioeconomic factors [10]. The authors found the incidences for depressive and eating disorders in 15–17-year-old females to be highest in the lowest SES group and vice versa; however, percentage increases between 2019 and 2021 were higher in the higher SES groups and lowest in the low SES group [10].

To our knowledge, the GIMD, a broad index covering various life domains, has not been used to examine potential differences in the occurrence of mental health disorders in children and adolescents. However, the British Index of Multiple Deprivation (IMD), on which the GIMD has been built, has been used, e.g., in the representative UK Household Longitudinal Study [35]. That study found that several inequalities narrowed during the pandemic with children in more advantaged groups experiencing greater deterioration in mental health, although this pattern was less pronounced for inequalities by sex and area deprivation [35].

In our analysis among adolescent girls no clear picture emerged. Despite a trend test for heterogeneity (year × GIMD interaction) being significant for anxiety disorders (but not for the other disorders), no consistent gradient across area-deprivation quintiles was observed for any of the disorders. However, these findings should be interpreted with caution. While they might suggest that the observed changes in mental health during the pandemic period occurred similarly across all socioeconomic contexts, our findings probably also indicate that area deprivation on a district level as provided by the GIMD might be outweighed by individual (socioeconomic) factors.

### 4.4. Strengths and Limitations

The main strengths of this study are the large database covering diagnoses from the in- and outpatient setting and the large time period allowing comparisons between pre-pandemic and pandemic time frames. The assessment of mental health was based on documented diagnoses which has the advantage that information is not biased by social desirability as compared to surveys but does not allow assessment of the severity of symptoms. It was further not possible to examine changes in service access during lockdowns, diagnostic delays or possible shifts from outpatient to inpatient/telemedicine care. As a further drawback, these administrative incidences and prevalences probably underestimate the true disease burden in the population since children who suffer from mental health disorders but do not seek or access healthcare services do not receive the respective diagnoses. Therefore, our findings do not necessarily reflect the need for psychotherapeutic care among children and adolescents. As an additional limitation, the pre-specified set of four disorders precluded broader analyses regarding general changes in mental health.

Further, the overall time period of 5 years with incidences calculated for 2019 to 2022 limits the ability to distinguish pandemic-period changes from pre-existing secular trends. A further limitation is attributed to the nature of the data with individual-level information (including insurance times) being only available for those children and adolescents living in Lower Saxony with at least one of the considered mental health diagnoses during the study period. Therefore, persons could have contributed to the incidence denominator without having been continuously insured during the previous year.

As the underlying data source lacked information about individual SES, the study aimed at assessing the influence of area deprivation by including the GIMD, a multidimensional index not solely addressing socioeconomic inequalities but also environmental factors or inadequate infrastructure. Its classification into quintiles (i.e., the quintiles derived from the German distribution) was chosen to ensure comparability to overall Germany. However, their uneven distribution in Lower Saxony resulted in two comparatively small marginal groups and as a consequence, wide confidence intervals hampered interpretability.

Last, this study is based on data from one SHI only, although it represents a high share of persons in the state of Lower Saxony. As insured individuals differ between SHIs with respect to, e.g., demographics, socioeconomic status or morbidity [36], this limits the generalizability of our results.

## 5. Conclusions

In this large German claims-based study, we observed an increase in the incidence of several mental health diagnoses as reflected by increased healthcare utilization among children and adolescents during the COVID-19 pandemic, particularly in 2021. Increases were most pronounced and consistent among adolescent girls. By contrast, no consistent gradient across levels of area deprivation was observed. Although prevalence changes were less evident and incidences declined somewhat in 2022, the persistence of elevated mental health diagnoses in certain subgroups underscores the necessity of providing targeted mental health support and monitoring long-term trends.

## Figures and Tables

**Table 1:** Trends in mental health diagnoses in children and adolescents stratified by sex and year.

	Incidence per 1000 Persons				Relative Risk		Prevalence per 1000 Persons					Prevalence Ratio	
Sex	2019	2020	2021	2022	2021 vs. 2019	2022 vs. 2019	2018	2019	2020	2021	2022	2021 vs. 2019	2022 vs. 2019
Depressive disorders													
All	13.3 (12.9–13.6)	12.2 (11.9–12.5)	13.7 (13.4–14.0)	11.9 (11.6–12.2)	1.03 (1.00–1.07)	0.90 (0.87–0.93)	26.2 (25.8–26.7)	26.5 (26.1–27.0)	25.1 (24.7–25.6)	26.2 (25.7–26.6)	24.2 (23.8–24.6)	0.99 (0.96–1.01)	0.91 (0.89–0.93)
Female	15.1 (14.6–15.6)	14.4 (13.9–14.9)	17.2 (16.7–17.7)	14.8 (14.4–15.3)	1.14 (1.09–1.19)	0.98 (0.94–1.03)	28.3 (27.6–29.0)	29.1 (28.4–29.7)	28.4 (27.7–29.0)	31.3 (30.6–32.0)	29.2 (28.6–29.8)	1.08 (1.04–1.11)	1.00 (0.97–1.04)
Male	11.6 (11.2–12.0)	10.1 (9.8–10.5)	10.5 (10.1–10.9)	9.1 (8.8–9.5)	0.90 (0.86–0.95)	0.79 (0.75–0.83)	24.3 (23.7–24.9)	24.2 (23.6–24.8)	22.1 (21.5–22.7)	21.3 (20.8–21.9)	19.4 (18.9–19.9)	0.88 (0.85–0.92)	0.80 (0.78–0.83)
Anxiety disorders													
All	32.6 (32.1–33.1)	32.1 (31.6–32.6)	34.7 (34.2–35.2)	30.1 (29.7–30.6)	1.06 (1.04–1.09)	0.92 (0.90–0.94)	65.0 (64.3–65.7)	65.5 (64.8–66.2)	63.8 (63.1–64.5)	66.0 (65.4–66.7)	60.3 (59.6–60.9)	1.01 (0.99–1.02)	0.92 (0.91–0.93)
Female	33.9 (33.1–34.6)	33.9 (33.2–34.6)	37.6 (36.9–38.4)	32.8 (32.1–33.5)	1.11 (1.08–1.14)	0.97 (0.94–1.00)	65.0 (64.0–66.0)	65.5 (64.5–66.5)	64.8 (63.9–65.8)	68.7 (67.7–69.7)	63.2 (62.3–64.1)	1.05 (1.03–1.07)	0.96 (0.94–0.98)
Male	31.4 (30.7–32.1)	30.5 (29.8–31.2)	32.0 (31.3–32.7)	27.7 (27.1–28.3)	1.02 (0.99–1.05)	0.88 (0.85–0.91)	64.9 (64.0–65.9)	65.5 (64.6–66.5)	62.9 (61.9–63.8)	63.5 (62.6–64.4)	57.5 (56.7–58.3)	0.97 (0.95–0.99)	0.88 (0.86–0.90)
ADHD													
All	16.3 (16.0–16.7)	16.3 (15.9–16.6)	18.0 (17.6–18.4)	16.1 (15.8–16.4)	1.10 (1.07–1.14)	0.98 (0.95–1.01)	56.7 (56.1–57.4)	54.5 (53.9–55.1)	53.2 (52.6–53.8)	54.6 (54.0–55.2)	50.4 (49.9–51.0)	1.00 (0.99–1.02)	0.92 (0.91–0.94)
Female	10.7 (10.3–11.1)	11.1 (10.6–11.5)	12.3 (11.9–12.8)	10.8 (10.5–11.2)	1.16 (1.10–1.22)	1.01 (0.96–1.07)	33.7 (33.0–34.4)	31.8 (31.1–32.5)	31.7 (31.0–32.4)	33.0 (32.3–33.7)	30.3 (29.7–30.9)	1.04 (1.01–1.07)	0.95 (0.92–0.98)
Male	21.9 (21.3–22.5)	21.4 (20.9–22.0)	23.6 (23.0–24.2)	21.2 (20.7–21.8)	1.08 (1.04–1.12)	0.97 (0.94–1.01)	78.2 (77.2–79.3)	75.8 (74.8–76.8)	73.4 (72.4–74.4)	75.0 (74.0–76.0)	69.4 (68.5–70.3)	0.99 (0.97–1.01)	0.92 (0.90–0.93)
Eating disorders													
All	2.3 (2.1–2.4)	2.3 (2.2–2.4)	2.7 (2.6–2.9)	2.2 (2.0–2.3)	1.21 (1.12–1.31)	0.96 (0.89–1.04)	4.6 (4.4–4.8)	4.0 (3.8–4.2)	3.9 (3.7–4.1)	4.4 (4.3–4.6)	3.9 (3.8–4.1)	1.11 (1.04–1.18)	0.97 (0.92–1.03)
Female	2.7 (2.5–2.9)	2.8 (2.6–3.1)	3.5 (3.2–3.7)	2.8 (2.6–3.0)	1.28 (1.15–1.41)	1.02 (0.92–1.13)	5.4 (5.1–5.7)	4.8 (4.6–5.1)	4.9 (4.6–5.2)	5.7 (5.4–6.0)	5.1 (4.8–5.4)	1.18 (1.09–1.27)	1.05 (0.97–1.14)
Male	1.8 (1.7–2.0)	1.8 (1.6–1.9)	2.0 (1.9–2.2)	1.6 (1.5–1.8)	1.13 (1.00–1.27)	0.88 (0.77–1.00)	3.8 (3.6–4.1)	3.2 (3.0–3.5)	3.0 (2.8–3.2)	3.3 (3.1–3.5)	2.8 (2.6–3.0)	1.01 (0.92–1.11)	0.86 (0.78–0.95)

## Data Availability

Restrictions apply to the availability of these data. Data were obtained from AOK Niedersachsen and were used under license for the current study and are not publicly available.

## Supplementary Materials

The following supporting information can be downloaded at: https://www.mdpi.com/article/10.3390/children13091245/s1, Supplemental Table S1: Distribution and characteristics of children and adolescents insured with AOK Lower Saxony by study year; Supplemental Table S2: Trends in mental health diagnoses in children and adolescents stratified by sex, age group and year; Supplemental Table S3: Trends in diagnoses of depressive disorders (narrow definition)* in children and adolescents stratified by sex, age group and year; Supplemental Figure S1: Prevalence of mental health diagnoses per 1000 children and adolescents stratified by sex, age group and year.
