# Supplementary material for: Trends in Mental Health Diagnoses Among Children and Adolescents Before and During the COVID-19 Pandemic in a Large German State
Source: Children (Basel). 2026 Sep 14;13(9):1245. doi: 10.3390/children13091245 (PMC13605553; doi:10.3390/children13091245)
Supplement: Supplementary file 1 [file children-13-01245-s001.zip › children-4511010-supplementary.pdf]

Trends in mental health diagnoses among children and adolescents before and during the COVID-19 pandemic in a large German state

Kathrin Jobski[1], Michael Dörks[1], Alexander M. Fassmer[1,2], Imke Meißner[3], Jona T. Stahmeyer[3], Sven Cornelisse[4], Luise Poustka[4], Christian J. Bachmann[5], Falk Hoffmann[1]

[1] Department of Health Services Research, Division of Outpatient Care and Pharmacoepidemiology, Carl von Ossietzky Universität Oldenburg, Oldenburg, Germany

[2] Institute for Clinical Epidemiology and Biometry, Julius-Maximilians-Universität Würzburg, Germany

[3] Health Services Research Unit, AOK Niedersachsen, Hannover, Germany

[4] Clinic for Child and Adolescent Psychiatry, Heidelberg University Hospital, Heidelberg, Germany

[5] Department of Child and Adolescent Psychiatry, University Hospital Ulm, Ulm, Germany

Supplemental information

Supplemental Table S1: Distribution and characteristics of children and adolescents insured with AOK Lower Saxony by study year

Study year	N	Females	Age group					GIMD quintile				
			0–5 years	6–8 years	9–11 years	12–14 years	15–17 years	Q1	Q2	Q3	Q4	Q5
2018	475,750	48.3%	33.8%	15.7%	16.3%	16.8%	17.5%	8.9%	23.1%	21.0%	40.3%	6.7%
2019	497,030	48.4%	34.7%	15.7%	16.1%	16.5%	17.1%	8.9%	23.2%	21.0%	40.2%	6.7%
2020	512,012	48.5%	35.2%	15.8%	15.8%	16.3%	16.9%	9.0%	23.3%	20.9%	40.2%	6.7%
2021	524,770	48.5%	35.5%	16.2%	15.7%	16.1%	16.6%	9.0%	23.3%	20.9%	40.2%	6.6%
2022	585,520	48.6%	37.0%	16.3%	15.3%	15.5%	15.9%	9.0%	23.3%	20.8%	40.3%	6.6%

GIMD: German Index of Multiple Deprivation

Supplemental Table S2: Trends in mental health diagnoses in children and adolescents stratified by sex, age group and year

		Incidence per 1000 persons				Relative risk		Prevalence per 1000 persons					Prevalence ratio	
Sex	Age	2019	2020	2021	2022	2021 vs. 2019	2022 vs. 2019	2018	2019	2020	2021	2022	2021 vs. 2019	2022 vs. 2019
**Depressive disorders**														
Female	0–5	3.1 (2.7-3.5)	3.2 (2.8-3.5)	2.8 (2.5-3.1)	2.5 (2.2-2.8)	0.91 (0.76-1.08)	0.81 (0.68-0.96)	6.2 (5.7-6.8)	6.0 (5.5-6.6)	5.9 (5.4-6.4)	5.4 (5.0-5.9)	4.6 (4.2-5.0)	0.90 (0.79-1.02)	0.77 (0.68-0.87)
	6–8	8.1 (7.3-9.1)	8.3 (7.4-9.2)	8.6 (7.7-9.5)	7.2 (6.4-8.0)	1.05 (0.91-1.23)	0.88 (0.75-1.03)	14.1 (12.9-15.4)	13.8 (12.7-15.1)	14.1 (13.0-15.3)	14.0 (12.9-15.2)	12.6 (11.6-13.6)	1.01 (0.90-1.14)	0.91 (0.81-1.02)
	9–11	12.7 (11.6-13.9)	11.3 (10.3-12.4)	12.7 (11.7-13.9)	10.4 (9.4-11.4)	1.00 (0.88-1.13)	0.81 (0.72-0.93)	24.6 (23.1-26.3)	25.8 (24.2-27.4)	23.0 (21.5-24.5)	23.9 (22.5-25.4)	20.5 (19.2-21.9)	0.93 (0.85-1.01)	0.80 (0.73-0.87)
	12–14	23.6 (22.1-25.2)	22.0 (20.6-23.5)	28.6 (27.0-30.2)	25.7 (24.3-27.3)	1.21 (1.11-1.32)	1.09 (1.00-1.19)	39.5 (37.6-41.5)	42.7 (40.8-44.8)	40.6 (38.7-42.6)	47.3 (45.3-49.4)	45.3 (43.4-47.3)	1.11 (1.04-1.18)	1.06 (1.00-1.13)
	15–17	41.1 (39.2-43.2)	40.8 (38.8-42.8)	51.5 (49.4-53.7)	47.4 (45.4-49.5)	1.25 (1.17-1.34)	1.15 (1.08-1.23)	77.6 (75.0-80.3)	80.5 (77.9-83.2)	82.5 (79.9-85.1)	95.4 (92.6-98.2)	97.0 (94.3-99.8)	1.18 (1.13-1.24)	1.20 (1.15-1.26)
Male	0–5	3.9 (3.5-4.3)	3.5 (3.1-3.9)	4.0 (3.6-4.4)	2.8 (2.5-3.1)	1.03 (0.89-1.19)	0.72 (0.62-0.84)	7.9 (7.3-8.5)	7.1 (6.6-7.7)	6.5 (6.0-7.0)	6.6 (6.1-7.1)	5.5 (5.0-5.9)	0.93 (0.83-1.04)	0.77 (0.69-0.86)
	6–8	11.3 (10.3-12.4)	10.3 (9.4-11.3)	10.2 (9.3-11.2)	8.7 (7.9-9.6)	0.91 (0.80-1.03)	0.77 (0.68-0.88)	20.2 (18.8-21.7)	20.5 (19.2-22.0)	18.9 (17.7-20.3)	18.4 (17.2-19.7)	16.9 (15.8-18.1)	0.90 (0.82-0.99)	0.82 (0.75-0.91)
	9–11	14.9 (13.8-16.2)	14.1 (13.0-15.3)	12.9 (11.8-14.0)	12.3 (11.3-13.3)	0.86 (0.77-0.97)	0.82 (0.73-0.92)	34.6 (32.9-36.5)	33.3 (31.7-35.1)	30.6 (29.0-32.3)	28.4 (26.9-30.0)	26.5 (25.1-28.0)	0.85 (0.79-0.92)	0.79 (0.74-0.86)
	12–14	15.2 (14.0-16.4)	13.2 (12.1-14.3)	14.2 (13.2-15.4)	12.7 (11.7-13.8)	0.94 (0.84-1.05)	0.84 (0.75-0.94)	34.8 (33.1-36.6)	36.1 (34.4-38.0)	33.6 (31.9-35.3)	32.1 (30.5-33.8)	30.0 (28.5-31.6)	0.89 (0.83-0.95)	0.83 (0.77-0.89)
	15–17	21.0 (19.7-22.4)	17.5 (16.3-18.8)	18.8 (17.5-20.1)	17.8 (16.7-19.1)	0.89 (0.81-0.98)	0.85 (0.77-0.93)	39.6 (37.8-41.5)	41.6 (39.7-43.5)	38.3 (36.6-40.2)	38.5 (36.8-40.4)	37.0 (35.4-38.8)	0.93 (0.87-0.99)	0.89 (0.84-0.95)
**Anxiety disorders**														
Female	0–5	21.9 (20.9-22.9)	22.0 (21.1-23.0)	22.0 (21.0-23.0)	18.0 (17.2-18.8)	1.00 (0.94-1.07)	0.82 (0.77-0.87)	36.1 (34.8-37.4)	34.2 (33.0-35.5)	33.6 (32.4-34.8)	33.2 (32.1-34.4)	27.3 (26.3-28.3)	0.97 (0.92-1.02)	0.80 (0.76-0.84)
	6–8	35.1 (33.3-37.1)	35.8 (34.0-37.7)	37.5 (35.7-39.5)	33.7 (32.0-35.4)	1.07 (0.99-1.15)	0.96 (0.89-1.03)	61.6 (59.2-64.1)	63.5 (61.1-66.0)	62.8 (60.5-65.3)	63.2 (60.9-65.6)	58.5 (56.4-60.7)	1.00 (0.94-1.05)	0.92 (0.87-0.97)
	9–11	36.8 (34.9-38.8)	33.6 (31.8-35.5)	38.3 (36.4-40.3)	33.9 (32.2-35.7)	1.04 (0.97-1.12)	0.92 (0.86-0.99)	77.0 (74.3-79.7)	80.2 (77.5-82.9)	74.0 (71.5-76.7)	77.0 (74.5-79.7)	70.9 (68.5-73.3)	0.96 (0.92-1.01)	0.88 (0.84-0.93)
	12–14	36.9 (35.0-38.8)	37.4 (35.5-39.4)	45.4 (43.4-47.6)	40.4 (38.5-42.3)	1.23 (1.15-1.32)	1.10 (1.02-1.18)	77.3 (74.7-80.0)	78.9 (76.3-81.6)	81.6 (78.9-84.3)	90.8 (88.0-93.6)	85.0 (82.4-87.6)	1.15 (1.10-1.20)	1.08 (1.03-1.13)
	15–17	51.9 (49.7-54.2)	54.1 (51.9-56.4)	64.0 (61.6-66.5)	59.5 (57.3-61.9)	1.23 (1.16-1.31)	1.15 (1.08-1.22)	101.7 (98.7-104.7)	104.9 (102.0-107.9)	107.6 (104.6-110.6)	121.2 (118.1-124.4)	123.8 (120.8-126.9)	1.16 (1.11-1.20)	1.18 (1.14-1.23)
Male	0–5	23.9 (22.9-24.9)	24.8 (23.8-25.8)	24.8 (23.8-25.8)	19.0 (18.2-19.8)	1.04 (0.98-1.10)	0.80 (0.75-0.84)	40.6 (39.3-42.0)	38.2 (37.0-39.5)	37.8 (36.6-39.1)	37.5 (36.3-38.7)	30.0 (29.0-31.0)	0.98 (0.94-1.03)	0.79 (0.75-0.82)
	6–8	43.4 (41.4-45.5)	41.3 (39.4-43.4)	44.7 (42.7-46.7)	39.8 (38.1-41.6)	1.03 (0.96-1.10)	0.92 (0.86-0.98)	79.4 (76.8-82.2)	80.4 (77.7-83.1)	76.3 (73.8-78.9)	77.8 (75.3-80.3)	72.3 (70.0-74.6)	0.97 (0.92-1.01)	0.90 (0.86-0.94)
	9–11	40.6 (38.7-42.7)	39.8 (37.8-41.8)	40.4 (38.5-42.5)	37.3 (35.5-39.1)	1.00 (0.93-1.07)	0.92 (0.86-0.98)	95.8 (92.9-98.7)	96.0 (93.2-98.9)	91.4 (88.6-94.2)	90.6 (87.9-93.4)	85.3 (82.8-87.9)	0.94 (0.90-0.98)	0.89 (0.85-0.93)
	12–14	30.6 (28.9-32.4)	27.2 (25.7-28.9)	30.2 (28.6-31.9)	28.1 (26.6-29.7)	0.99 (0.91-1.07)	0.92 (0.85-0.99)	75.8 (73.2-78.4)	81.3 (78.7-83.9)	77.0 (74.5-79.6)	77.8 (75.3-80.3)	74.6 (72.3-77.0)	0.96 (0.91-1.00)	0.92 (0.88-0.96)
	15–17	28.0 (26.5-29.6)	26.8 (25.3-28.4)	28.8 (27.3-30.5)	26.1 (24.7-27.6)	1.03 (0.95-1.12)	0.93 (0.86-1.01)	60.0 (57.8-62.3)	63.3 (61.0-65.6)	61.9 (59.8-64.2)	65.7 (63.5-68.1)	62.3 (60.2-64.5)	1.04 (0.99-1.09)	0.99 (0.94-1.04)

		Incidence per 1000 persons				Relative risk		Prevalence per 1000 persons					Prevalence ratio	
Sex	Age	2019	2020	2021	2022	2021 vs. 2019	2022 vs. 2019	2018	2019	2020	2021	2022	2021 vs. 2019	2022 vs. 2019
**ADHD**														
Female	0–5	5.4 (4.9-5.9)	6.5 (6.0-7.0)	6.6 (6.1-7.2)	5.5 (5.1-6.0)	1.23 (1.09-1.39)	1.02 (0.90-1.15)	8.6 (8.0-9.3)	8.0 (7.4-8.6)	8.6 (8.0-9.2)	8.8 (8.3-9.5)	7.4 (6.9-7.9)	1.11 (1.00-1.23)	0.93 (0.83-1.03)
	6–8	23.0 (21.5-24.6)	21.8 (20.4-23.4)	24.0 (22.5-25.5)	20.5 (19.2-21.9)	1.04 (0.95-1.14)	0.89 (0.81-0.98)	48.8 (46.6-51.1)	44.1 (42.0-46.2)	42.0 (40.1-44.0)	43.0 (41.1-45.0)	39.0 (37.3-40.8)	0.98 (0.91-1.04)	0.89 (0.83-0.94)
	9–11	17.4 (16.1-18.8)	18.1 (16.8-19.5)	19.0 (17.6-20.4)	17.7 (16.5-19.0)	1.09 (0.98-1.21)	1.02 (0.92-1.13)	62.8 (60.4-65.3)	60.2 (57.9-62.7)	59.9 (57.6-62.3)	61.1 (58.8-63.5)	56.6 (54.5-58.8)	1.01 (0.96-1.07)	0.94 (0.89-0.99)
	12–14	8.8 (7.9-9.8)	8.9 (8.0-9.9)	10.4 (9.4-11.4)	9.4 (8.5-10.3)	1.18 (1.02-1.37)	1.07 (0.92-1.24)	44.7 (42.6-46.8)	45.1 (43.1-47.2)	44.1 (42.1-46.1)	45.7 (43.7-47.8)	43.5 (41.7-45.5)	1.01 (0.95-1.08)	0.97 (0.91-1.03)
	15–17	6.0 (5.3-6.8)	6.2 (5.5-7.0)	9.2 (8.4-10.2)	8.5 (7.7-9.4)	1.54 (1.31-1.81)	1.42 (1.20-1.67)	31.0 (29.4-32.8)	29.6 (28.0-31.3)	31.8 (30.2-33.5)	36.1 (34.3-37.9)	36.7 (35.0-38.5)	1.22 (1.13-1.31)	1.24 (1.15-1.33)
Male	0–5	11.0 (10.3-11.7)	11.5 (10.8-12.2)	12.2 (11.5-12.9)	10.2 (9.6-10.8)	1.10 (1.01-1.20)	0.93 (0.85-1.01)	18.0 (17.1-18.9)	16.6 (15.7-17.4)	16.8 (16.0-17.6)	17.1 (16.3-17.9)	14.6 (13.9-15.3)	1.03 (0.96-1.10)	0.88 (0.82-0.95)
	6–8	50.1 (47.9-52.3)	45.7 (43.7-47.9)	51.0 (48.9-53.1)	46.5 (44.6-48.5)	1.02 (0.96-1.08)	0.93 (0.87-0.99)	108.7 (105.6-111.8)	104.4 (101.4-107.4)	95.9 (93.1-98.8)	100.9 (98.1-103.8)	94.2 (91.7-96.8)	0.97 (0.93-1.01)	0.90 (0.87-0.94)
	9–11	35.9 (34.0-37.8)	36.9 (35.0-38.9)	39.3 (37.3-41.3)	34.6 (32.9-36.4)	1.10 (1.02-1.18)	0.96 (0.90-1.04)	143.8 (140.4-147.3)	140.5 (137.2-143.9)	138.1 (134.8-141.4)	139.0 (135.7-142.3)	128.3 (125.3-131.4)	0.99 (0.96-1.02)	0.91 (0.88-0.94)
	12–14	18.8 (17.5-20.3)	17.7 (16.5-19.1)	19.4 (18.1-20.8)	19.8 (18.5-21.1)	1.03 (0.93-1.14)	1.05 (0.95-1.16)	112.0 (109.0-115.1)	110.4 (107.5-113.4)	109.0 (106.1-112.0)	110.4 (107.5-113.4)	110.7 (107.9-113.6)	1.00 (0.96-1.04)	1.00 (0.97-1.04)
	15–17	9.9 (9.0-10.9)	10.2 (9.3-11.2)	12.2 (11.2-13.3)	11.2 (10.3-12.3)	1.23 (1.08-1.40)	1.13 (0.99-1.29)	74.1 (71.7-76.6)	75.2 (72.8-77.7)	75.3 (72.9-77.8)	78.6 (76.1-81.1)	73.6 (71.3-76.0)	1.04 (1.00-1.09)	0.98 (0.94-1.02)
**Eating disorders**														
Female	0–5	2.6 (2.3-3.0)	2.1 (1.8-2.5)	2.1 (1.9-2.5)	1.7 (1.5-2.0)	0.81 (0.67-0.98)	0.64 (0.53-0.78)	4.1 (3.7-4.6)	3.9 (3.5-4.3)	3.1 (2.8-3.5)	3.1 (2.7-3.5)	2.7 (2.4-3.0)	0.80 (0.68-0.94)	0.69 (0.59-0.81)
	6–8	1.5 (1.1-1.9)	1.5 (1.1-1.9)	1.8 (1.4-2.2)	1.3 (1.0-1.7)	1.18 (0.83-1.68)	0.89 (0.62-1.28)	2.7 (2.2-3.3)	2.4 (2.0-3.0)	2.2 (1.8-2.7)	2.7 (2.2-3.3)	2.4 (2.0-2.9)	1.11 (0.84-1.46)	0.98 (0.74-1.29)
	9–11	1.7 (1.3-2.1)	1.8 (1.5-2.3)	2.0 (1.6-2.5)	1.4 (1.1-1.8)	1.17 (0.85-1.63)	0.82 (0.58-1.16)	3.3 (2.8-4.0)	2.7 (2.3-3.3)	3.1 (2.6-3.8)	3.3 (2.8-3.9)	2.4 (2.0-2.9)	1.20 (0.93-1.55)	0.88 (0.67-1.15)
	12–14	3.2 (2.7-3.8)	3.5 (3.0-4.2)	4.4 (3.8-5.1)	4.1 (3.6-4.8)	1.37 (1.09-1.72)	1.28 (1.02-1.61)	6.4 (5.6-7.2)	5.5 (4.8-6.3)	5.8 (5.1-6.6)	7.1 (6.3-8.0)	6.8 (6.1-7.7)	1.29 (1.08-1.54)	1.25 (1.05-1.48)
	15–17	4.5 (3.9-5.2)	5.9 (5.2-6.7)	8.5 (7.6-9.4)	6.8 (6.1-7.6)	1.90 (1.59-2.27)	1.52 (1.26-1.83)	11.3 (10.3-12.4)	10.4 (9.5-11.5)	11.8 (10.8-12.9)	15.3 (14.1-16.5)	14.5 (13.4-15.7)	1.46 (1.30-1.65)	1.39 (1.23-1.57)
Male	0–5	2.4 (2.1-2.8)	2.3 (2.0-2.6)	2.5 (2.2-2.9)	1.8 (1.6-2.1)	1.04 (0.86-1.25)	0.76 (0.63-0.92)	4.7 (4.3-5.2)	3.6 (3.2-4.0)	3.2 (2.9-3.6)	3.3 (3.0-3.7)	2.7 (2.4-3.0)	0.93 (0.80-1.09)	0.76 (0.65-0.90)
	6–8	1.6 (1.3-2.1)	1.1 (0.8-1.4)	1.8 (1.5-2.3)	1.5 (1.2-1.8)	1.12 (0.81-1.56)	0.90 (0.64-1.26)	3.2 (2.7-3.8)	3.1 (2.6-3.6)	2.1 (1.7-2.6)	2.9 (2.5-3.5)	2.4 (2.0-2.9)	0.97 (0.75-1.24)	0.79 (0.61-1.01)
	9–11	1.5 (1.2-1.9)	2.0 (1.7-2.5)	1.8 (1.4-2.3)	1.5 (1.2-1.9)	1.22 (0.87-1.70)	0.98 (0.70-1.39)	3.8 (3.3-4.5)	3.0 (2.5-3.6)	3.3 (2.8-3.9)	3.2 (2.7-3.8)	2.6 (2.2-3.1)	1.06 (0.83-1.35)	0.87 (0.68-1.12)
	12–14	1.3 (1.0-1.7)	1.7 (1.4-2.2)	2.1 (1.7-2.6)	1.5 (1.2-1.9)	1.58 (1.13-2.20)	1.13 (0.80-1.61)	3.7 (3.2-4.4)	3.0 (2.6-3.6)	3.3 (2.8-3.9)	3.8 (3.3-4.4)	3.2 (2.8-3.8)	1.25 (0.99-1.57)	1.07 (0.84-1.35)
	15–17	1.6 (1.2-2.0)	1.2 (0.9-1.6)	1.5 (1.1-1.9)	1.4 (1.1-1.8)	0.93 (0.66-1.31)	0.92 (0.66-1.28)	2.9 (2.5-3.5)	3.2 (2.7-3.7)	2.7 (2.3-3.2)	3.0 (2.5-3.5)	3.1 (2.6-3.6)	0.95 (0.75-1.20)	0.97 (0.77-1.23)

Supplemental Table S3: Trends in diagnoses of depressive disorders (narrow definition)* in children and adolescents stratified by sex, age group and year

		Incidence per 1000 persons				Relative risk		Prevalence per 1000 persons					Prevalence ratio	
**Sex**	**Age**	**2019**	**2020**	**2021**	**2022**	**2021 vs. 2019**	**2022 vs. 2019**	**2018**	**2019**	**2020**	**2021**	**2022**	**2021 vs. 2019**	**2022 vs. 2019**
All		5.1 (4.9-5.3)	4.9 (4.7-5.1)	6.0 (5.8-6.2)	5.3 (5.1-5.5)	1.18 (1.12-1.25)	1.04 (0.99-1.09)	8.5 (8.3-8.8)	8.9 (8.6-9.1)	8.7 (8.4-8.9)	9.8 (9.5-10.1)	9.4 (9.2-9.7)	1.10 (1.06-1.15)	1.06 (1.02-1.10)
Female		6.9 (6.6-7.3)	7.0 (6.7-7.4)	9.0 (8.6-9.3)	7.9 (7.6-8.3)	1.29 (1.21-1.38)	1.14 (1.07-1.22)	11.4 (11.0-11.9)	12.2 (11.7-12.6)	12.3 (11.9-12.8)	14.7 (14.2-15.2)	14.3 (13.8-14.7)	1.21 (1.15-1.27)	1.17 (1.12-1.23)
Male		3.3 (3.1-3.6)	2.9 (2.7-3.1)	3.2 (3.0-3.4)	2.8 (2.6-3.0)	0.96 (0.88-1.06)	0.83 (0.76-0.91)	5.8 (5.6-6.2)	5.8 (5.5-6.1)	5.2 (5.0-5.5)	5.2 (4.9-5.5)	4.9 (4.6-5.1)	0.90 (0.83-0.97)	0.84 (0.78-0.90)
Female	0–5	0.2 (0.2-0.4)	0.2 (0.1-0.4)	0.2 (0.1-0.3)	0.1 (0.1-0.2)	0.69 (0.36-1.35)	0.40 (0.19-0.85)	0.3 (0.2-0.4)	0.3 (0.2-0.4)	0.3 (0.2-0.4)	0.2 (0.1-0.3)	0.1 (0.1-0.2)	0.70 (0.39-1.28)	0.48 (0.25-0.90)
	6–8	0.7 (0.5-1.1)	0.9 (0.6-1.2)	0.9 (0.7-1.3)	0.7 (0.5-0.9)	1.28 (0.79-2.08)	0.90 (0.54-1.51)	0.9 (0.6-1.2)	1.0 (0.7-1.4)	1.1 (0.8-1.5)	1.2 (0.9-1.6)	0.9 (0.7-1.2)	1.19 (0.78-1.81)	0.90 (0.58-1.40)
	9–11	2.4 (2.0-2.9)	2.5 (2.0-3.0)	2.6 (2.2-3.2)	2.2 (1.8-2.6)	1.09 (0.83-1.44)	0.90 (0.67-1.19)	3.2 (2.7-3.8)	3.8 (3.2-4.5)	3.6 (3.1-4.3)	3.7 (3.1-4.3)	3.1 (2.6-3.7)	0.97 (0.77-1.21)	0.82 (0.65-1.04)
	12–14	11.2 (10.2-12.3)	11.7 (10.7-12.8)	16.4 (15.2-17.7)	15.0 (13.9-16.2)	1.46 (1.30-1.65)	1.34 (1.19-1.51)	16.3 (15.1-17.6)	17.3 (16.1-18.6)	17.0 (15.8-18.3)	22.8 (21.4-24.3)	22.4 (21.1-23.9)	1.32 (1.19-1.45)	1.30 (1.18-1.43)
	15–17	27.2 (25.7-28.9)	27.6 (26.1-29.3)	35.5 (33.7-37.3)	33.8 (32.1-35.5)	1.30 (1.21-1.41)	1.24 (1.15-1.34)	46.3 (44.2-48.4)	50.0 (47.9-52.1)	51.9 (49.8-54.1)	61.7 (59.5-64.1)	64.4 (62.2-66.7)	1.24 (1.17-1.31)	1.29 (1.22-1.36)
Male	0–5	0.2 (0.1-0.3)	0.2 (0.1-0.3)	0.2 (0.1-0.3)	0.1 (0.1-0.2)	0.78 (0.40-1.52)	0.59 (0.30-1.18)	0.3 (0.2-0.5)	0.3 (0.2-0.4)	0.3 (0.2-0.4)	0.2 (0.1-0.3)	0.2 (0.1-0.3)	0.77 (0.42-1.40)	0.63 (0.34-1.16)
	6–8	1.2 (0.9-1.6)	1.0 (0.7-1.3)	0.9 (0.7-1.2)	0.8 (0.5-1.0)	0.75 (0.49-1.13)	0.61 (0.40-0.94)	1.4 (1.0-1.8)	1.5 (1.2-2.0)	1.3 (1.0-1.7)	1.1 (0.8-1.4)	1.0 (0.7-1.3)	0.70 (0.48-1.03)	0.63 (0.43-0.92)
	9–11	2.3 (1.9-2.8)	2.6 (2.2-3.2)	2.7 (2.2-3.2)	2.4 (2.0-2.9)	1.16 (0.88-1.53)	1.03 (0.78-1.35)	4.6 (3.9-5.3)	4.1 (3.6-4.8)	3.9 (3.4-4.6)	3.6 (3.1-4.2)	3.7 (3.2-4.3)	0.87 (0.70-1.09)	0.89 (0.72-1.10)
	12–14	4.6 (4.0-5.3)	4.7 (4.1-5.4)	4.7 (4.1-5.4)	4.3 (3.7-4.9)	1.03 (0.84-1.25)	0.93 (0.76-1.13)	8.8 (8.0-9.8)	8.6 (7.8-9.5)	8.5 (7.7-9.4)	8.0 (7.2-8.9)	7.7 (6.9-8.5)	0.93 (0.80-1.07)	0.89 (0.77-1.03)
	15–17	11.3 (10.3-12.3)	9.0 (8.2-10.0)	11.1 (10.1-12.1)	9.8 (9.0-10.7)	0.98 (0.87-1.11)	0.87 (0.77-0.99)	18.6 (17.4-19.9)	19.5 (18.3-20.8)	17.4 (16.2-18.6)	18.6 (17.4-19.9)	18.0 (16.8-19.2)	0.96 (0.87-1.05)	0.92 (0.84-1.01)

* The narrow definition for depressive disorders included diagnoses of depressive episode (F32-) or recurrent depressive disorder (F33-).

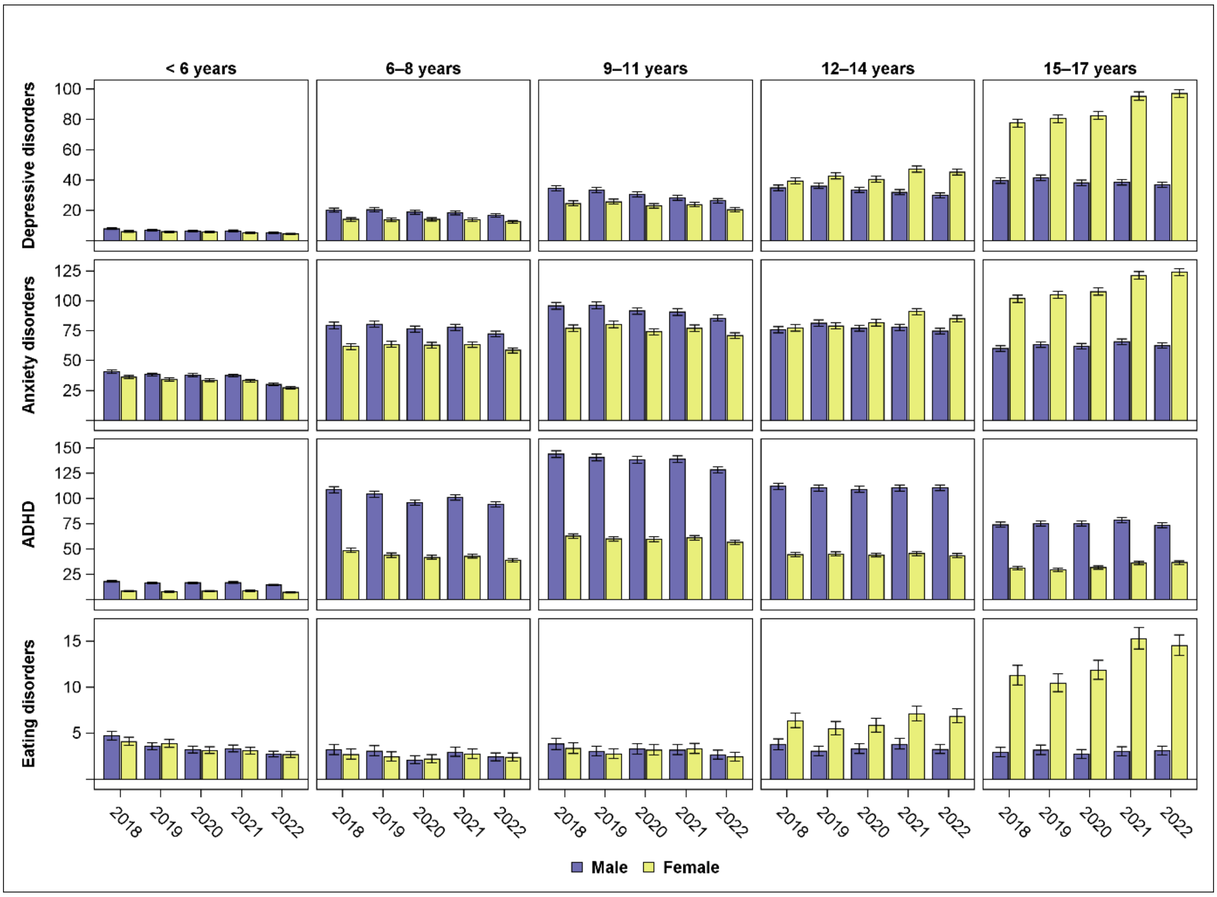

Supplemental Figure S1: Prevalence of mental health diagnoses per 1000 children and adolescents stratified by sex, age group and year

Note: The scales of the axes differ.
